# Bitwise XOR linear space creation through permutation and the ease to enable XOR-free processes

**DOI:** 10.1038/s41598-025-17542-9

**Published:** 2025-10-02

**Authors:** Surendra Shetty, Radhakrishna Dodmane, Niranjan N. Prabhu, Nagaraja Shetty

**Affiliations:** 1 Department of Master of Computer Applications, NMAM Institute of Technology, NITTE (Deemed to be University), Nitte, Karnataka 574110 India; 2 Department of Computer and Communication Engineering, NMAM Institute of Technology, NITTE (Deemed to be University), Nitte, Karnataka 574110 India; 3 Department of Mechanical and Industrial Engineering, Manipal Institute of Technology, Manipal Academy of Higher Education, Manipal, Karnataka 576104 India

**Keywords:** SNOW 3G, SNOW 5G, Permutation, XOR-free, Computational science, Computer science

## Abstract

The speed of operation execution is receiving more emphasis in contemporary computing settings. Because the time-consuming unit operations may be processed more quickly through the right modifications on the same, which would be a more attractive feature for applications like data transfer and cryptography. Hence, a new software-oriented technique is suggested in this study for usage in contexts where faster executions are necessary. The XOR logical operation are accelerated by the suggested way. This work first describes how to efficiently build the XOR linear space via permutation of values. In the present article, the method of designing the XOR linear space is elaborated to assist XOR-free processes/operations. The regular key stream generating scheme SNOW 3G and SNOW 5G is used in conjunction with the proposed bitwise XOR-free procedure/operations for the empirical evidence. The average speed-up gained using the projected XOR-Free process/operations for SNOW 3G is 1.38× and for SNOW 5G is 1.0193×. Similarly, the throughput attained is 459.82 Kbps and $23.659\text{ Mbps}$ for SNOW 5G and SNOW 3G, respectively.

## Introduction

The speed of execution of any unit operations significantly affects performance in current computing environments. As the operation speed increases, the overall processing would be faster, which would greatly impact the performance system under consideration^1–4^. However, the major concern the researchers must address is that the attempt to make the change should not affect the actual operation of the unit under consideration. The output effect of the same unit operations must also be safeguarded.

The speed of unit operations does have effects in almost every area of computer science. Many researchers have been working towards finding solutions to speed up unit operations^1^. Cryptography or cybersecurity is one of the areas where execution speed has a significant impact. So, improving the unit operation’s speed will have a direct impact on how well cryptographic algorithms perform as a whole. So, the main criteria in the field of computation, especially in cryptography, are discovering the answers or adequate techniques to raise the efficiency of unit operations and, therefore, the performance speed of the entire algorithm. Keeping these ideas in mind, the study is disposed to suggest potential methods to increase the execution speed. Following a thorough investigation in this direction, we have decided to focus on logical operations like bitwise XOR.

From the literature study, it was observed that logical bitwise XOR operations are frequently used in conventional symmetric key cryptography systems^1,5,6^. According to^1,6–8^, the repeated XOR operations cause delays in the total execution time because they must be loaded and stored in the cache repeatedly. Eliminating bitwise XOR operations is one way to reduce the latency caused by these load and store processes. Yet doing away with bitwise XOR would directly impact how effective and efficient the algorithms are as a whole. Hence, the solutions must minimize the execution speed, whereas the impact of the XOR is retained. Thus, the study was carried out in the direction of proposing an XOR-free operation to retain all the effects and efficiency of the algorithms under consideration. This could be achieved through a Look-Up-Table (LUT), also known as XOR linear space.

The LUTs/XOR linear space is constructed well in advance to store the possible outcomes of bitwise XOR operations. Many mathematical models have been proposed to construct LUTs^1,5^, which are highlighted at the end of subsection “An example construction of XOR linear space based on the proposed permutation function”, represented with an example. This research suggests a brand-new mathematical framework for creating bitwise XOR LUTs as a backup option. Subsequently, this LUT is used to enable XOR-free operations. The proposed model is implemented in symmetric stream ciphers, SNOW 3G^1^ and SNOW 5G^9^. This bitwise XOR LUT is created far in advance of the SNOW 3G and SNOW 5G XOR operations.

## Background

As the study progresses, it’s observed that the XOR-free operations would accelerate the execution speed^8^. This opens the door for the work to dive into the other possible impacts of implementing the XOR-free. The outcome of the same is presented below.

As mentioned earlier, bitwise XOR-free operations could be implemented by fetching and placing the outcome of XOR operations (from the LUT), which is required to be constructed well in advance. Fetch and place values of XOR operation outcomes from LUTs exhibit increased efficiency in the computation system, especially boosting the execution speed^8^. This is also helpful in lowering the consumption of power compared to the hardware implementation^10^, as it limited copying operations. Lower consumption power is a great advantage in cryptography, even in other areas of computation^10^. These features of XOR-free implementation through LUTs have gained large attention in the computing environment. Various works on XOR-free implementations are briefly presented next.

Dodmani et al.^1^ elaborates on a new mathematical model for the construction of an XOR linear space based on Pauli’s matrix efficiently by a recursive approach. They observed that the theoretical time complexity of the same is $O\left( {\log_{2} \left( {\frac{n}{{2^{n} }}} \right)} \right)$. The constructed XOR linear space is implemented on stream cipher techniques, such as SNOW 3G, to enable XOR-free computations. The suggested XOR-free methods are implemented in lieu of the evidential demonstration using common SNOW 3G key stream generation schemes. Their experimental results show that the XOR Free techniques on the SNOW 3G have an average speed of 1.36×, and the associated throughput is 21.84 Mbps.

A reliable technique to lower the computation cost of the bitwise XOR free by a circular condition has been put forth by Naseri et al.^11^. The methods of estimating bitwise XOR gates without additional expense are revealed in the work of^11–13^ on the XOR-free techniques. Nasari et al.’s^11^ novel FleXOR technique to increase the overall computation speed is based on the XOR-free method. Chu et al.’s^14^ XOR methods/operations are structurally rewritten, and LUTs and rules of optimization have drastically reduced the XOR operations. Amaru et al.^15^ proposed on enhancing the efficiency of XOR operations, which is the base for the findings of^14^.

The innovative method developed by Vandierendonck et al.^16^ to carry out bitwise XOR operations in an improved manner helps to eliminate cache misses and CPU load. The bitwise XOR preparation developed by Luo et al.^17^ significantly improves the performance over traditional methods. Similar to this, Narayana et al.’s^18^ modification of the Boolean function into the fixed polarity of Reed-Muller form enabled the efficient blending of bitwise XOR trees with the least amount of detectable switching activity. Their research findings show that compared to traditional methods, there are respectable power savings. Nikoubin et al.^19^ developed a novel approach for low-voltage bitwise XOR circuits. This would benefit the energy optimization needed for the XOR method/operations. An alternative technique has been put out by Purohit et al.^20^ to create an XOR-free design that uses a significant reduction in dynamic power. The recommended approach by^20^ facilitates the removal of XOR treatment of the quasi-feed-forward generating polynomial of the convolution encoder and lowers the complexity of logical operators. With less complexity in the design, this approach reduces hardware price by approximately 15% and dynamic power by approximately 21.4%. From the mechanisms that have been proposed so far by various researchers^1,10,20^, it was decided to propose new mathematical answers in the same direction to expedite the process.

The proposed endeavour aims to investigate the potential for enhancing the efficiency enhancements associated with bitwise XOR operations. It explores the possibility of achieving these enhancements through the use of software molds. To achieve this, this work introduces a novel mathematical approach that aims to incorporate XOR-free procedures in addition to traditional XOR operations. By focusing on building a comprehensive XOR linear space, the present work seeks to facilitate more efficient computational methods, ultimately enhancing the overall performance of algorithms that rely on bitwise operations.

It would be suitable if the anticipated approach were verified against some system in terms of execution speedup. In order to calculate the speedup gained as a result of its implementation, the suggested approach has been verified on industry-standard SNOW 3G and SNOW 5G systems. Understanding the standard SNOW 3G and SNOW 5G stream cipher scheme is important before implementing the suggested bitwise XOR-free methods. Hence, a brief discussion about the operation of the SNOW 3G and SNOW 5G stream cipher systems is mentioned below.

A 128-bit initialization linear (IV) stream and a 128-bit key are used to control the regular SNOW 3G stream cipher system^21–23^, which creates a steady stream of 32-bit words and features a traditional word-oriented stream cipher structure. The five parts of the conventional SNOW-3G process are a Finite State Machine (FSM) with S-boxes, S1 and S2, a Linear Feedback Shift Register (LFSR), MULa, MULxPOW, and DIVa. This cipher operates in two main modes: one in which the key initialization process is completed, and the other in which the cipher method is completed without generating any output. After that, the cipher runs in key-generation mode. An input word $F$ of 32 bits, a finite state machine’s output is collected by the linear feedback shift register (LFSR) in initialization mode (FSM). Each of the 16 internal stages in the LFSR can hold a word of size 32 bits. While in keystream mode, the LFSR is designed to reject input data.

Ekdahl et al.^24^ established a new associate with the standard SNOW cipher system family with the intention of encountering very high-speed encryption called SNOW 5. The offered SNOW 5 is estimated to be appropriate for the forthcoming mobile communication 5G. The proposed standard SNOW 5 cipher system is the revised SNOW 3G architecture with the accelerating AES instruction and modern CPU’s capability to accomplish the large integers. The improved LFSR layer runs eight times faster than the FSM layer. New 128-bit registers are added in place of 32-bit registers in the FSM layer with the addition of two AES encryptions of a single round. The initialization of standard SNOW 5 comprises masking by keystream bits at the end. The proposed method by^5,24^ is much faster than AES–256 without compromising the security.

With this understanding, the following section delivers a thorough description of the various mathematical solutions proposed to construct linear XOR space, various mechanisms implemented to increase the speed of execution associated with the XOR operation, and the algorithm considered for its implementation. The XOR linear space constructed consists of the resultant values of XOR operations between two operands. The same would be used as a replacement for the XOR operations to enable XOR-free computation.

## Methodology

It has been demonstrated through the work of numerous researchers that XOR-free operations would significantly speed up the execution of any algorithms. It has been intended to make XOR-free computation easier by keeping the overall impact of XOR operations, based on the literature investigation. Only by retrieving and placing resultant values from a pre-compiled list or linear space is this possible. The creation of the XOR linear space in advance is, therefore, difficult in this situation. In this study, a mathematical framework is put forth to build the linear space that will house the output values of XOR calculations. The construction of the XOR linear space is then described in detail.

### Pictorial representation of the proposed permutation method

Before proceeding to the actual model, it is preferable to consider the possible elements of the XOR linear space for any given set of operands. Hence, this paper starts with an XOR linear space that holds the possible values of XOR operations in a specific position for specific operands. The pictorial representation of the XOR linear space is presented in the Table 1,

**Table 1:** XOR Linear Space of 8*8 (where i and j are in the range from 0 to 7).

XOR linear space								
${\oplus }_{{\varvec{i}},{\varvec{j}}}$	0	1	2	3	4	5	6	7
0	0	1	2	3	4	5	6	7
1	1	0	3	2	5	4	7	6
2	2	3	0	1	6	7	4	5
3	3	2	1	0	7	6	5	4
4	4	5	6	7	0	1	2	3
5	5	4	7	6	1	0	3	2
6	6	7	4	5	2	3	0	1
7	7	6	5	4	3	2	1	0

where $\oplus_{{{\varvec{i}},{\varvec{j}}}}$ denotes the XOR operation for the *i*th and *j*th operands.

The first row and the first column in Table 1 represent the operands of the XOR method/operations that are indicated by the indices of the XOR linear space. Whereas the XOR process/operations between any two operands are denoted using the symbol $\oplus_{{{\varvec{i}},{\varvec{j}}}}$, where i and j represent the operands of rows and columns, respectively. The first row, other than the indices in Table 1, is the resultant value of the XOR between 0 and (0–7) in sequence. Similarly, the next row is XOR between 1 and (0–7), then between 2 and (0–7), 3 and (0–7), and so on. The last row represents the XOR between 7 and (0–7).

The careful, sustained interpretation of the position of the values in the Table 1 exhibits the properties of permutations. That is, elements of all other rows are purely the rearrangement of elements of the first row (i.e., XOR of 0 and (0–7) in some specific pattern. The careful analysis of these patterns led this work to model the mathematical solutions that could permute the first row to generate any row denoting $i \oplus j$, where $i,j = 0,1,2, \ldots upto\;n$.

Since the values of the Table 1 exhibits a specific pattern, the work starts by pairing/grouping two consecutive values from 0 to $n$ with the label C_n_, where n = 0,1,2,3, etc. The paired values are termed as tuples with respect to Table 1 and are shown in Table 2, where $$n = 1,{ }C_{1} = \left[ {\begin{array}{*{20}c} 0 & 1 \\ \end{array} } \right]$$, similarly, when $${\text{n}} = 2,{\text{ C}}_{2} = \left[ {\begin{array}{*{20}c} 2 & 3 \\ \end{array} } \right]$$ and so on. Based on these expressions or tuples C_1_, C_2_, C_3_, etc., and the rest of the rows of the XOR linear space could be generated using the required pattern. The row ${\mathbf{R}}_{{{\mathbf{i}} = 0}}$ is a sequence of tuples $C_{1} ,C_{2} ,C_{3}$. Similarly, the ${\mathbf{R}}_{{{\mathbf{i}} = 1}}$ denotes the circular shift (left or right) of the tuples $C_{1} ,C_{2} ,C_{3}$ That is $\ll C_{1} , \ll C_{2} , \ll C_{3} \ldots$. The row ${\mathbf{R}}_{{{\mathbf{i}} = 2}}$ is the swapping of tuples $C_{1} { },C_{2}$ and $C_{3} ,C_{4}$ and so on. All other rows are permuted; accordingly, the same is presented in the Table 2 as expressions.

**Table 2:** Proposed permutation method by expression for 8*8 XOR Linear Space.

Proposed permutation to generate XOR linear space				
Rows	$$C_{1} = \left[ {\begin{array}{*{20}c} 0 & 1 \\ \end{array} } \right]$$	$$C_{2} = \left[ {\begin{array}{*{20}c} 2 & 3 \\ \end{array} } \right]$$	$$C_{3} = \left[ {\begin{array}{*{20}c} 4 & 5 \\ \end{array} } \right]$$	$$C_{4} = \left[ {\begin{array}{*{20}c} 6 & 7 \\ \end{array} } \right]$$
${\mathbf{R}}_{{{\mathbf{i}} = 0}}$	$C_{1}$	$C_{2}$	$C_{3}$	$C_{4}$
${\mathbf{R}}_{{{\mathbf{i}} = 1}}$	$\ll C_{1}$	$\ll C_{2}$	$\ll C_{3}$	$\ll C_{4}$
${\mathbf{R}}_{{{\mathbf{i}} = 2}}$	$C_{2}$	$C_{1}$	$C_{4}$	$C_{3}$
${\mathbf{R}}_{{{\mathbf{i}} = 3}}$	$\ll C_{2}$	$\ll C_{1}$	$\ll C_{4}$	$\ll C_{3}$
${\mathbf{R}}_{{{\mathbf{i}} = 4}}$	$C_{3}$	$C_{4}$	$C_{1}$	$C_{2}$
${\mathbf{R}}_{{{\mathbf{i}} = 5}}$	$\ll C_{3}$	$\ll C_{4}$	$\ll C_{1}$	$\ll C_{2}$
${\mathbf{R}}_{{{\mathbf{i}} = 6}}$	$C_{4}$	$C_{3}$	$C_{2}$	$C_{1}$
${\mathbf{R}}_{{{\mathbf{i}} = 7}}$	$\ll C_{4}$	$\ll C_{3}$	$\ll C_{2}$	$\ll C_{1}$

The mathematical solutions for generating the tuples ${\text{C}}_{{\text{n}}} ,{\text{where}}\;{\text{n}} = 0,1,2,{\text{etc}}$ and the corresponding permuted tuples are shown in the Table 3 as a tuple of values. In the Table 3, the first row shows the mathematical equation that would generate any tuple ${\text{C}}_{{\text{n}}}$ for any value of n = 0,1, 2, etc. The Table 2 shows the mathematical expressions for every element of the XOR linear space, whereas the Table 3 denotes the corresponding tuples with respect to Table 2.

**Table 3:** Proposed Permutation method by values of expression for 8*8 XOR Linear Space.

$${\text{C}}_{{\text{n}}} = \left\{ {\left[ {2\left( {{\text{n}} - 1} \right) + \left[ {\begin{array}{*{20}c} 0 & 1 \\ \end{array} } \right]} \right]|\forall \left( {{\text{n}} > 0 \& \left( {{\text{i}} = 0} \right)} \right)} \right\}$$				
Rows	$${\text{C}}_{1} = \left[ {\begin{array}{*{20}c} 0 & 1 \\ \end{array} } \right]$$	$${\text{C}}_{2} = \left[ {\begin{array}{*{20}c} 2 & 3 \\ \end{array} } \right]$$	$${\text{C}}_{3} = \left[ {\begin{array}{*{20}c} 4 & 5 \\ \end{array} } \right]$$	$${\text{C}}_{4} = \left[ {\begin{array}{*{20}c} 6 & 7 \\ \end{array} } \right]$$
${\mathbf{R}}_{{{\mathbf{i}} = 0}}$	$$\left[ {\begin{array}{*{20}c} 0 & 1 \\ \end{array} } \right]$$	$$\left[ {\begin{array}{*{20}c} 2 & 3 \\ \end{array} } \right]$$	$$\left[ {\begin{array}{*{20}c} 4 & 5 \\ \end{array} } \right]$$	$$\left[ {\begin{array}{*{20}c} 6 & 7 \\ \end{array} } \right]$$
${\mathbf{R}}_{{{\mathbf{i}} = 1}}$	$$\left[ {\begin{array}{*{20}c} 1 & 0 \\ \end{array} } \right]$$	$$\left[ {\begin{array}{*{20}c} 3 & 2 \\ \end{array} } \right]$$	$$\left[ {\begin{array}{*{20}c} 5 & 4 \\ \end{array} } \right]$$	$$\left[ {\begin{array}{*{20}c} 7 & 6 \\ \end{array} } \right]$$
${\mathbf{R}}_{{{\mathbf{i}} = 2}}$	$$\left[ {\begin{array}{*{20}c} 2 & 3 \\ \end{array} } \right]$$	$$\left[ {\begin{array}{*{20}c} 0 & 1 \\ \end{array} } \right]$$	$$\left[ {\begin{array}{*{20}c} 6 & 7 \\ \end{array} } \right]$$	$$\left[ {\begin{array}{*{20}c} 4 & 5 \\ \end{array} } \right]$$
${\mathbf{R}}_{{{\mathbf{i}} = 3}}$	$$\left[ {\begin{array}{*{20}c} 3 & 2 \\ \end{array} } \right]$$	$$\left[ {\begin{array}{*{20}c} 1 & 0 \\ \end{array} } \right]$$	$$\left[ {\begin{array}{*{20}c} 7 & 6 \\ \end{array} } \right]$$	$$\left[ {\begin{array}{*{20}c} 5 & 4 \\ \end{array} } \right]$$
${\mathbf{R}}_{{{\mathbf{i}} = 4}}$	$$\left[ {\begin{array}{*{20}c} 4 & 5 \\ \end{array} } \right]$$	$$\left[ {\begin{array}{*{20}c} 6 & 7 \\ \end{array} } \right]$$	$$\left[ {\begin{array}{*{20}c} 0 & 1 \\ \end{array} } \right]$$	$$\left[ {\begin{array}{*{20}c} 2 & 3 \\ \end{array} } \right]$$
${\mathbf{R}}_{{{\mathbf{i}} = 5}}$	$$\left[ {\begin{array}{*{20}c} 5 & 4 \\ \end{array} } \right]$$	$$\left[ {\begin{array}{*{20}c} 7 & 6 \\ \end{array} } \right]$$	$$\left[ {\begin{array}{*{20}c} 1 & 0 \\ \end{array} } \right]$$	$$\left[ {\begin{array}{*{20}c} 3 & 2 \\ \end{array} } \right]$$
${\mathbf{R}}_{{{\mathbf{i}} = 6}}$	$$\left[ {\begin{array}{*{20}c} 6 & 7 \\ \end{array} } \right]$$	$$\left[ {\begin{array}{*{20}c} 4 & 5 \\ \end{array} } \right]$$	$$\left[ {\begin{array}{*{20}c} 2 & 3 \\ \end{array} } \right]$$	$$\left[ {\begin{array}{*{20}c} 0 & 1 \\ \end{array} } \right]$$
${\mathbf{R}}_{{{\mathbf{i}} = 7}}$	$$\left[ {\begin{array}{*{20}c} 7 & 6 \\ \end{array} } \right]$$	$$\left[ {\begin{array}{*{20}c} 5 & 4 \\ \end{array} } \right]$$	$$\left[ {\begin{array}{*{20}c} 3 & 2 \\ \end{array} } \right]$$	$$\left[ {\begin{array}{*{20}c} 1 & 0 \\ \end{array} } \right]$$

Table 4 represents both the tuple of values from Table 2 and the mathematical expressions from Table 3, which would efficiently construct the XOR linear space. Every row of Table 4 is either a permutation/rearrangement of values within the tuple Cn or a permutation among a group of tuples **C**_**n**_. The comprehensive description of how to permute to generate an XOR linear space is furnished as a mathematical model below. As said earlier, every row of the XOR linear space has a specific pattern, and it’s purely the rearrangement of a tuple of values of the first row. The careful, sustained analysis of these patterns made this work to develop mathematical models for generating a specific row pattern of XOR linear space. The mathematical models are presented below with examples for each.

**Table 4:** Proposed permutation method by expressions and the corresponding values for 8*8 XOR linear space.

$${\text{C}}_{{\text{n}}} = \left\{ {\left[ {2\left( {{\text{n}} - 1} \right) + \left[ {\begin{array}{*{20}c} 0 & 1 \\ \end{array} } \right]} \right]|\forall \left( {{\text{n}} > 0 \& \left( {{\text{i}} = 0} \right)} \right)} \right\}$$				
Rows	$${\text{C}}_{1} = \left[ {\begin{array}{*{20}c} 0 & 1 \\ \end{array} } \right]$$	$${\text{C}}_{2} = \left[ {\begin{array}{*{20}c} 2 & 3 \\ \end{array} } \right]$$	$${\text{C}}_{3} = \left[ {\begin{array}{*{20}c} 4 & 5 \\ \end{array} } \right]$$	$${\text{C}}_{4} = \left[ {\begin{array}{*{20}c} 6 & 7 \\ \end{array} } \right]$$
${\mathbf{R}}_{{{\mathbf{i}} = 0}}$	$${\text{C}}_{1} = \left[ {\begin{array}{*{20}c} 0 & 1 \\ \end{array} } \right]$$	$${\text{C}}_{2} = \left[ {\begin{array}{*{20}c} 2 & 3 \\ \end{array} } \right]$$	$${\text{C}}_{3} = \left[ {\begin{array}{*{20}c} 4 & 5 \\ \end{array} } \right]$$	$${\text{C}}_{4} = \left[ {\begin{array}{*{20}c} 6 & 7 \\ \end{array} } \right]$$
${\mathbf{R}}_{{{\mathbf{i}} = 1}}$	$$\ll C_{1} = \left[ {\begin{array}{*{20}c} 1 & 0 \\ \end{array} } \right]$$	$$\ll C_{2} = \left[ {\begin{array}{*{20}c} 3 & 2 \\ \end{array} } \right]$$	$$\ll C_{3} = \left[ {\begin{array}{*{20}c} 5 & 4 \\ \end{array} } \right]$$	$$\ll C_{4} = \left[ {\begin{array}{*{20}c} 7 & 6 \\ \end{array} } \right]$$
${\mathbf{R}}_{{{\mathbf{i}} = 2}}$	$${\text{C}}_{2} = \left[ {\begin{array}{*{20}c} 2 & 3 \\ \end{array} } \right]$$	$${\text{C}}_{1} = \left[ {\begin{array}{*{20}c} 0 & 1 \\ \end{array} } \right]$$	$${\text{C}}_{4} = \left[ {\begin{array}{*{20}c} 6 & 7 \\ \end{array} } \right]$$	$${\text{C}}_{3} = \left[ {\begin{array}{*{20}c} 4 & 5 \\ \end{array} } \right]$$
${\mathbf{R}}_{{{\mathbf{i}} = 3}}$	$$\ll C_{2} = \left[ {\begin{array}{*{20}c} 3 & 2 \\ \end{array} } \right]$$	$$\ll C_{1} = \left[ {\begin{array}{*{20}c} 1 & 0 \\ \end{array} } \right]$$	$$\ll C_{4} = \left[ {\begin{array}{*{20}c} 7 & 6 \\ \end{array} } \right]$$	$$\ll C_{3} = \left[ {\begin{array}{*{20}c} 5 & 4 \\ \end{array} } \right]$$
${\mathbf{R}}_{{{\mathbf{i}} = 4}}$	$${\text{C}}_{3} = \left[ {\begin{array}{*{20}c} 4 & 5 \\ \end{array} } \right]$$	$${\text{C}}_{4} = \left[ {\begin{array}{*{20}c} 6 & 7 \\ \end{array} } \right]$$	$${\text{C}}_{1} = \left[ {\begin{array}{*{20}c} 0 & 1 \\ \end{array} } \right]$$	$${\text{C}}_{2} = \left[ {\begin{array}{*{20}c} 2 & 3 \\ \end{array} } \right]$$
${\mathbf{R}}_{{{\mathbf{i}} = 5}}$	$$\ll C_{3} = \left[ {\begin{array}{*{20}c} 5 & 4 \\ \end{array} } \right]$$	$$\ll C_{4} = \left[ {\begin{array}{*{20}c} 7 & 6 \\ \end{array} } \right]$$	$$\ll C_{1} = \left[ {\begin{array}{*{20}c} 1 & 0 \\ \end{array} } \right]$$	$$\ll C_{2} = \left[ {\begin{array}{*{20}c} 3 & 2 \\ \end{array} } \right]$$
${\mathbf{R}}_{{{\mathbf{i}} = 6}}$	$${\text{C}}_{4} = \left[ {\begin{array}{*{20}c} 6 & 7 \\ \end{array} } \right]$$	$${\text{C}}_{3} = \left[ {\begin{array}{*{20}c} 4 & 5 \\ \end{array} } \right]$$	$${\text{C}}_{2} = \left[ {\begin{array}{*{20}c} 2 & 3 \\ \end{array} } \right]$$	$${\text{C}}_{1} = \left[ {\begin{array}{*{20}c} 0 & 1 \\ \end{array} } \right]$$
${\mathbf{R}}_{{{\mathbf{i}} = 7}}$	$$\ll C_{4} = \left[ {\begin{array}{*{20}c} 7 & 6 \\ \end{array} } \right]$$	$$\ll C_{3} = \left[ {\begin{array}{*{20}c} 5 & 4 \\ \end{array} } \right]$$	$$\ll C_{2} = \left[ {\begin{array}{*{20}c} 3 & 2 \\ \end{array} } \right]$$	$$\ll C_{1} = \left[ {\begin{array}{*{20}c} 1 & 0 \\ \end{array} } \right]$$

### Proposed permutation models to construct the XOR linear space.

The XOR linear space as Tables 2, 3 and 4 shown above are constructed based on the equations from (1) to (6). Every tuple of an XOR linear space is a pair of elements, which is constructed using the Eq. (1). The first row of the XOR linear space is a sequence of tuples ***C***_***n***_, where ***n = 1, 2, 3, etc.,*** and ***R***_***i***_** = 0** denotes the first row. The second row can be generated either by performing a circular shift on every tuple of Cn or by using Eq. (2).

1 $$\sigma^{i = 0} = R_{i = 0} = C_{n} = \left\{ {\left[ {2\left( {n - 1} \right) + \left[ {\begin{array}{*{20}c} 0 & 1 \\ \end{array} } \right]} \right]|\forall \left( {n > 0 \& \left( {i = 0} \right)} \right)} \right\}$$

Equation (2) is similar to Eq. (1), but the tuple of values considered for the operations is swapped. The output of this operation results in a pair of values that are circular shifts of the tuples generated using the Eq. (1), which is denoted as the expression $\ll C_{n}$.

2 $$\sigma^{i = 1} = R_{i = 1} = C_{n} = \left\{ {\left[ {2\left( {n - 1} \right) + \left[ {\begin{array}{*{20}c} 1 & 0 \\ \end{array} } \right]} \right]|\forall \left( {n > 0 \& \left( {i = 0} \right)} \right)} \right\} or R_{i = 1} = \ll C_{n}$$

These two equations, that is, (1) and (2), are considered the base for constructing all other rows of the XOR linear space. Since there are dependencies among all the rows of XOR linear space, this work concentrated on proposing the mathematical solutions based on the row indices, such as the power of 2 (i.e., **2**^**r**^**,** where **r = 1, 2, 3, etc.)** and its next row. The Eq. (3) generates all the rows of the XOR linear space, where its indices are equal to **2**^**r**^. The symbol $||$ is to denote the concatenation of tuples **C**_**n**_ as per the outcomes of the XOR operations. In these rows, the circular shift would be carried out only among the tuples **C**_**n**_ but not within the tuple.

3 $$\sigma^{{\left( {\forall \left( {i = 2^{r} } \right)} \right)}} = \left\{ {||_{{\left[ {j = j + 2^{{\left( {{\raise0.7ex\hbox{$i$} \!\mathord{\left/ {\vphantom {i 2}}\right.\kern-0pt} \!\lower0.7ex\hbox{$2$}}} \right)}} } \right]}}^{\left[ \infty \right]} \left( { \ll_{{\left[ {\left( {2^{{\left( {\left( {{\raise0.7ex\hbox{$i$} \!\mathord{\left/ {\vphantom {i 2}}\right.\kern-0pt} \!\lower0.7ex\hbox{$2$}}} \right) - 1} \right)}} } \right)C_{n} } \right]}} \left( {||_{{\left[ {n = j} \right]}}^{{\left[ {n < \left( {j + 2^{{\left( {{\raise0.7ex\hbox{$i$} \!\mathord{\left/ {\vphantom {i 2}}\right.\kern-0pt} \!\lower0.7ex\hbox{$2$}}} \right)}} } \right)} \right]}} \left( {C_{n} } \right) } \right)} \right)|\left( {j = 1} \right) \& \left( {\forall \left( {i = 2^{r} } \right)} \right) } \right\}$$

Similarly, Eq. (4) is designed to develop all the elements of the XOR linear space, where its row indices are **2**^**r**^** + 1**. These rows depend on Eq. (1), and the circular shift is carried out both between and within the tuples Cn.

4 $$\sigma^{{\left( {\forall \left( {i = 2^{r} + 1} \right)} \right)}} = \left\{ {||_{{\left[ {j = j + 2^{{\left( {{\raise0.7ex\hbox{$i$} \!\mathord{\left/ {\vphantom {i 2}}\right.\kern-0pt} \!\lower0.7ex\hbox{$2$}}} \right)}} } \right]}}^{\left[ \infty \right]} \left( { \ll_{{\left[ {\left( {2^{{\left( {\left( {{\raise0.7ex\hbox{$i$} \!\mathord{\left/ {\vphantom {i 2}}\right.\kern-0pt} \!\lower0.7ex\hbox{$2$}}} \right) - 1} \right)}} } \right)\left( { \ll C_{n} } \right)} \right]}} \left( {|| _{{\left[ {n = j} \right]}}^{{\left[ {n < \left( {j + 2^{{\left( {{\raise0.7ex\hbox{$i$} \!\mathord{\left/ {\vphantom {i 2}}\right.\kern-0pt} \!\lower0.7ex\hbox{$2$}}} \right)}} } \right)} \right]}} \left( { \ll C_{n} } \right) } \right)} \right)|\left( {j = 1} \right) \& \left( {\forall \left( {i = 2^{r} + 1} \right)} \right)} \right\}$$

The rest of the even-indexed rows of XOR linear space, other than the row indices **2**^**r**^ are computed using Eq. (5) below, which is dependent on Eq. (3). Here, in this equation, the circular shift is carried out among the tuples **C**_**n**_.

5 $$\begin{aligned} R_{i} & = \left\{ { \ll_{{\left[ {\left( {2^{k} } \right)C_{n} } \right]}} \left( {\sigma^{{\left( {2^{r} } \right)}} } \right){|}\forall } \right.\left( {\left( {k = \left( {{\raise0.7ex\hbox{${\left( {2^{{\left( {r - l} \right)}} } \right)}$} \!\mathord{\left/ {\vphantom {{\left( {2^{{\left( {r - l} \right)}} } \right)} 2}}\right.\kern-0pt} \!\lower0.7ex\hbox{$2$}}} \right) - 1} \right)} \right.,\left( {l = 1,2,3, \ldots upto \left( {r - l} \right) = 1} \right) \\ & \quad \left. {\left. {\& \left( {i\;is\;even} \right)\& \left( {2^{r} < i < \left( {2^{{\left( {r + 1} \right)}} } \right)} \right)} \right)} \right\} \\ \end{aligned}$$

Similarly, the rest of the odd-indexed rows of XOR linear space, other than the row indices (**2**^**r**^** + 1),** are computed through Eq. (6), which is dependent on Eq. (4).

6 $$\begin{aligned} R_{i} & = \left\{ { \ll_{{\left[ {\left( {2^{k} } \right)C_{n} } \right]}} \left( {\sigma^{{\left( {2^{r} + 1} \right)}} } \right){|}\forall } \right.\left( {\left( {k = \left( {{\raise0.7ex\hbox{${\left( {2^{{\left( {r - l} \right)}} } \right)}$} \!\mathord{\left/ {\vphantom {{\left( {2^{{\left( {r - l} \right)}} } \right)} 2}}\right.\kern-0pt} \!\lower0.7ex\hbox{$2$}}} \right) - 1} \right)} \right.,\left( {l = 1,2,3, \ldots upto\left( {r - l} \right) = 1} \right) \\ & \quad \left. {\left. {\& \left( {i\;is\;odd} \right)\& \left( {2^{r} < i < \left( {2^{{\left( {r + 1} \right)}} } \right)} \right)} \right)} \right\} \\ \end{aligned}$$

The equations from (2) to (6) listed above are all permutation operations; hence, computation costs are negligible. Only Eq. (1) exhibits the scalar additions depending on the number of tuples to be generated. This XOR linear space will be built much more before the start of the XOR process/operations in the algorithms under consideration. Then, these values from the XOR linear space are required to be fetched and placed as a result of XOR operations to facilitate XOR-free. The meticulous description of the above equations for the construction of elements of the XOR linear space is presented below with an example, which considers the linear space of 8*8.

### An example construction of XOR linear space based on the proposed permutation function.

Let us assume the XOR linear space size is *m* = *2*^*r*^ = *2*^*3*^ = *8*. That is, the total order of the linear space is *m* × *m* (*m* rows and *m* columns).

Below is an example of creating the various rows as per the permutation equations.

1. The first row of the XOR linear space when *i* = *0* is created as follows:
  $$R_{i = 0} = C_{n} = \left\{ {\left[ {2\left( {n - 1} \right) + \left[ {\begin{array}{*{20}c} 0 & 1 \\ \end{array} } \right]} \right]|\forall \left( {\left( {n > 0} \right) \& \left( {i = 0} \right)} \right)} \right\}$$
  When $$n = 1,{ }C_{1} = \left\{ {\left[ {2\left( {1 - 1} \right) + \left[ {\begin{array}{*{20}c} 0 & 1 \\ \end{array} } \right]} \right]} \right\} = \left\{ {\left[ {0 + \left[ {\begin{array}{*{20}c} 0 & 1 \\ \end{array} } \right]} \right]} \right\} = \left\{ {\left[ {\begin{array}{*{20}c} 0 & 1 \\ \end{array} } \right]} \right\}$$
  Similarly, $$C_{2} = \left\{ {\left[ {\begin{array}{*{20}c} 2 & 3 \\ \end{array} } \right]} \right\}$$, $$C_{3} = \left\{ {\left[ {\begin{array}{*{20}c} 4 & 5 \\ \end{array} } \right]} \right\}$$ and $$C_{4} = \left\{ {\left[ {\begin{array}{*{20}c} 6 & 7 \\ \end{array} } \right]} \right\}$$.
2. The second row of the XOR linear space when *i* = *1* is created as follows:
  $$R_{i = 1} = \ll C_{n}$$
  When $$n = 1,{ } \ll C_{1} = \ll \left\{ {\left[ {2\left( {1 - 1} \right) + \left[ {\begin{array}{*{20}c} 0 & 1 \\ \end{array} } \right]} \right]} \right\} = \ll \left\{ {\left[ {0 + \left[ {\begin{array}{*{20}c} 0 & 1 \\ \end{array} } \right]} \right]} \right\} = \left\{ {\left[ {\begin{array}{*{20}c} 1 & 0 \\ \end{array} } \right]} \right\}$$.
  Similarly, $$\ll C_{2} = \left\{ {\left[ {\begin{array}{*{20}c} 3 & 2 \\ \end{array} } \right]} \right\}$$, $$\ll C_{3} = \left\{ {\left[ {\begin{array}{*{20}c} 5 & 4 \\ \end{array} } \right]} \right\}$$ and $$\ll C_{4} = \left\{ {\left[ {\begin{array}{*{20}c} 7 & 6 \\ \end{array} } \right]} \right\}$$.
3. The third row of the XOR linear space when *i *= 2 is created as follows:
  $$\begin{aligned} & \sigma ^{{\left( {\forall ~\left( {i = 2^{r} } \right)} \right)}} = \left\{ { \ll _{{\left[ {\left( {2^{{\left( {\left( {{\raise0.7ex\hbox{$i$} \!\mathord{\left/ {\vphantom {i 2}}\right.\kern-\nulldelimiterspace} \!\lower0.7ex\hbox{$2$}}} \right) - 1} \right)}} } \right)C_{n} } \right]}} \left( {||_{{\left[ {n = j} \right]}}^{{\left[ {n < \left( {j + 2^{{\left( {{\raise0.7ex\hbox{$i$} \!\mathord{\left/ {\vphantom {i 2}}\right.\kern-\nulldelimiterspace} \!\lower0.7ex\hbox{$2$}}} \right)}} } \right)} \right]}} \left( {C_{n} } \right)~} \right)} \right.~ \\ & \quad \left. {\left| {\left( {\left( {j = 1} \right)\;and\;incremented\left( {j = j + 2^{{\left( {{\raise0.7ex\hbox{$i$} \!\mathord{\left/ {\vphantom {i 2}}\right.\kern-\nulldelimiterspace} \!\lower0.7ex\hbox{$2$}}} \right)}} } \right)} \right)} \right.\& \left( {\forall ~\left( {i = 2^{r} } \right)} \right)} \right\} \\ \end{aligned}$$
  When *j* = *1*:
    $$n = 1 :C_{1} = \left\{ {\left[ {\begin{array}{*{20}c} 0 & 1 \\ \end{array} } \right]} \right\}$$
    $$n = 2 :C_{2} = \left\{ {\left[ {\begin{array}{*{20}c} 2 & 3 \\ \end{array} } \right]} \right\}$$
  When concatenated $C_{1} \& C_{2}$ at the first level:
    $$C_{1} ||C_{2} = \left\{ {\left[ {\begin{array}{*{20}c} 0 & 1 \\ \end{array} } \right]\left[ {\begin{array}{*{20}c} 2 & 3 \\ \end{array} } \right]} \right\}$$
  Left circular rotation of $C_{1} \& C_{2}$:
    $$C_{2} ||C_{1} = \left\{ {\left[ {\begin{array}{*{20}c} 2 & 3 \\ \end{array} } \right]\left[ {\begin{array}{*{20}c} 0 & 1 \\ \end{array} } \right]} \right\}$$
  When *j* = 3:
    $$n = 3 :C_{3} = \left\{ {\left[ {\begin{array}{*{20}c} 4 & 5 \\ \end{array} } \right]} \right\}$$
    $$n = 4 :C_{4} = \left\{ {\left[ {\begin{array}{*{20}c} 6 & 7 \\ \end{array} } \right]} \right\}$$
  When concatenated $C_{3} \& C_{4}$ at the first level:
    $$C_{3} ||C_{4} = \left\{ {\left[ {\begin{array}{*{20}c} 4 & 5 \\ \end{array} } \right]\left[ {\begin{array}{*{20}c} 6 & 7 \\ \end{array} } \right]} \right\}$$
  Left circular rotation of $C_{3} \& C_{4}$:
    $$C_{4} ||C_{3} = \left\{ {\left[ {\begin{array}{*{20}c} 6 & 7 \\ \end{array} } \right]\left[ {\begin{array}{*{20}c} 4 & 5 \\ \end{array} } \right]} \right\}$$
  When concatenated $C_{1} , C_{2} , C_{3} \& C_{4}$ at the second/final level after the rotation at the first level:
    $$C_{2} \left| {\left| {C_{1} } \right|} \right|C_{4} ||C_{3} = \left\{ {\left[ {\begin{array}{*{20}c} 2 & 3 \\ \end{array} } \right]\left[ {\begin{array}{*{20}c} 0 & 1 \\ \end{array} } \right]} \right\}\left\{ {\left[ {\begin{array}{*{20}c} 6 & 7 \\ \end{array} } \right]\left[ {\begin{array}{*{20}c} 4 & 5 \\ \end{array} } \right]} \right\}$$
4. Similarly, for the fourth row $R_{i = 1} = \ll C_{n}$ is considered as a base row tuple. That is the fourth row of the XOR linear space when *i* = *3* is created as follows:
  $$\begin{aligned} & \sigma ^{{\left( {\forall ~\left( {i = 2^{r} + 1} \right)} \right)}} = \left\{ { \ll _{{\left[ {\left( {2^{{\left( {\left( {{\raise0.7ex\hbox{$i$} \!\mathord{\left/ {\vphantom {i 2}}\right.\kern-\nulldelimiterspace} \!\lower0.7ex\hbox{$2$}}} \right) - 1} \right)}} } \right) \ll C_{n} } \right]}} \left( {||_{{\left[ {n = j} \right]}}^{{\left[ {n < \left( {j + 2^{{\left( {{\raise0.7ex\hbox{$i$} \!\mathord{\left/ {\vphantom {i 2}}\right.\kern-\nulldelimiterspace} \!\lower0.7ex\hbox{$2$}}} \right)}} } \right)} \right]}} \left( { \ll C_{n} } \right)~} \right)} \right. \\ & \quad \left. {~\left| {\left( {\left( {j = 1} \right)\;and\;incremented\left( {j = j + 2^{{\left( {{\raise0.7ex\hbox{$i$} \!\mathord{\left/ {\vphantom {i 2}}\right.\kern-\nulldelimiterspace} \!\lower0.7ex\hbox{$2$}}} \right)}} } \right)} \right)\& \left( {\forall ~\left( {i = 2^{r} + 1} \right)} \right)} \right.} \right\} \\ \end{aligned}$$
  When *j* = *1*:
    $$n = 1 : \ll C_{1} = \left\{ {\left[ {\begin{array}{*{20}c} 1 & 0 \\ \end{array} } \right]} \right\}$$
    $$n = 2 : \ll C_{2} = \left\{ {\left[ {\begin{array}{*{20}c} 3 & 2 \\ \end{array} } \right]} \right\}$$
  When concatenated $\ll C_{1} \& \ll C_{2}$ at the first level:
    $$\ll C_{1} || \ll C_{2} = \left\{ {\left[ {\begin{array}{*{20}c} 1 & 0 \\ \end{array} } \right]\left[ {\begin{array}{*{20}c} 3 & 2 \\ \end{array} } \right]} \right\}$$
  Left circular rotation of $\ll C_{1} \& \ll C_{2}$:
    $$\ll C_{2} || \ll C_{1} = \left\{ {\left[ {\begin{array}{*{20}c} 3 & 2 \\ \end{array} } \right]\left[ {\begin{array}{*{20}c} 1 & 0 \\ \end{array} } \right]} \right\}$$
  When *j* = *3*:
    $$n = 3 : \ll C_{3} = \left\{ {\left[ {\begin{array}{*{20}c} 5 & 4 \\ \end{array} } \right]} \right\}$$
    $$n = 4 : \ll C_{4} = \left\{ {\left[ {\begin{array}{*{20}c} 7 & 6 \\ \end{array} } \right]} \right\}$$
  When concatenated $\ll C_{3} \& \ll C_{4}$ at the first level:
    $$\ll C_{3} || \ll C_{4} = \left\{ {\left[ {\begin{array}{*{20}c} 5 & 4 \\ \end{array} } \right]\left[ {\begin{array}{*{20}c} 7 & 6 \\ \end{array} } \right]} \right\}$$
  Left circular rotation of $\ll C_{3} \& \ll C_{4}$:
    $$\ll C_{4} || \ll C_{3} = \left\{ {\left[ {\begin{array}{*{20}c} 7 & 6 \\ \end{array} } \right]\left[ {\begin{array}{*{20}c} 5 & 4 \\ \end{array} } \right]} \right\}$$
  When concatenated $\ll C_{1} , \ll C_{2} , \ll C_{3} \& \ll C_{4}$ at the second/final level after the rotation at the first level:
    $$\ll C_{2} \left| {\left| { \ll C_{1} } \right|} \right| \ll C_{4} || \ll C_{3} = \left\{ {\left[ {\begin{array}{*{20}c} 3 & 2 \\ \end{array} } \right]\left[ {\begin{array}{*{20}c} 1 & 0 \\ \end{array} } \right]} \right\}\left\{ {\left[ {\begin{array}{*{20}c} 7 & 6 \\ \end{array} } \right]\left[ {\begin{array}{*{20}c} 5 & 4 \\ \end{array} } \right]} \right\}$$
5. The fifth row of the XOR linear space when *i* = *4* is created as follows:
  $$\begin{aligned} & \sigma ^{{\left( {\forall ~\left( {i = 2^{r} } \right)} \right)}} = \left\{ { \ll _{{\left[ {\left( {2^{{\left( {\left( {{\raise0.7ex\hbox{$i$} \!\mathord{\left/ {\vphantom {i 2}}\right.\kern-\nulldelimiterspace} \!\lower0.7ex\hbox{$2$}}} \right) - 1} \right)}} } \right)C_{n} } \right]}} \left( {||_{{\left[ {n = j} \right]}}^{{\left[ {n < \left( {j + 2^{{\left( {{\raise0.7ex\hbox{$i$} \!\mathord{\left/ {\vphantom {i 2}}\right.\kern-\nulldelimiterspace} \!\lower0.7ex\hbox{$2$}}} \right)}} } \right)} \right]}} \left( {C_{n} } \right)~} \right)~} \right. \\ & \quad \left. {\left| {\left( {\left( {j = 1} \right)\;and\;incremented\left( {j = j + 2^{{\left( {{\raise0.7ex\hbox{$i$} \!\mathord{\left/ {\vphantom {i 2}}\right.\kern-\nulldelimiterspace} \!\lower0.7ex\hbox{$2$}}} \right)}} } \right)} \right)\& \left( {\forall ~\left( {i = 2^{r} } \right)} \right)} \right.} \right\} \\ \end{aligned}$$
  When *j* = *1*:
    $$n = 1:C_{n} = \left\{ {\left[ {\begin{array}{*{20}c} 0 & 1 \\ \end{array} } \right]} \right\}$$
    $$n = 2:C_{n} = \left\{ {\left[ {\begin{array}{*{20}c} 2 & 3 \\ \end{array} } \right]} \right\}$$
    $$n = 3:C_{n} = \left\{ {\left[ {\begin{array}{*{20}c} 4 & 5 \\ \end{array} } \right]} \right\}$$
    $$n = 4:C_{n} = \left\{ {\left[ {\begin{array}{*{20}c} 6 & 7 \\ \end{array} } \right]} \right\}$$
  When concatenated $C_{1} , C_{2} , C_{3} \& C_{4}$ at the first level:
    $$C_{1} \left| {\left| {C_{2} } \right|} \right|C_{3} ||C_{4} = \left\{ {\left[ {\begin{array}{*{20}c} 0 & 1 \\ \end{array} } \right]\left[ {\begin{array}{*{20}c} 2 & 3 \\ \end{array} } \right]\left[ {\begin{array}{*{20}c} 4 & 5 \\ \end{array} } \right]\left[ {\begin{array}{*{20}c} 6 & 7 \\ \end{array} } \right]} \right\}$$
  Left circular rotation of two-row tuples on the concatenation is such that:
    $$C_{3} \left| {\left| {C_{4} } \right|} \right|C_{1} ||C_{2} = \left\{ {\left[ {\begin{array}{*{20}c} 4 & 5 \\ \end{array} } \right]\left[ {\begin{array}{*{20}c} 6 & 7 \\ \end{array} } \right]\left[ {\begin{array}{*{20}c} 0 & 1 \\ \end{array} } \right]\left[ {\begin{array}{*{20}c} 2 & 3 \\ \end{array} } \right]} \right\}$$
6. The sixth row of the XOR linear space when *i* = *5* is created as follows:
  $$\begin{aligned} & \sigma ^{{\left( {\forall ~\left( {i = 2^{r} + 1} \right)} \right)}} = \left\{ { \ll _{{\left[ {\left( {2^{{\left( {\left( {{\raise0.7ex\hbox{$i$} \!\mathord{\left/ {\vphantom {i 2}}\right.\kern-\nulldelimiterspace} \!\lower0.7ex\hbox{$2$}}} \right) - 1} \right)}} } \right) \ll C_{n} } \right]}} \left( {||_{{\left[ {n = j} \right]}}^{{\left[ {n < \left( {j + 2^{{\left( {{\raise0.7ex\hbox{$i$} \!\mathord{\left/ {\vphantom {i 2}}\right.\kern-\nulldelimiterspace} \!\lower0.7ex\hbox{$2$}}} \right)}} } \right)} \right]}} \left( { \ll C_{n} } \right)~} \right)} \right. \\ & \quad \left. {\left| {\left( {\left( {j = 1} \right)\;and\;incremented\;\left( {j = j + 2^{{\left( {{\raise0.7ex\hbox{$i$} \!\mathord{\left/ {\vphantom {i 2}}\right.\kern-\nulldelimiterspace} \!\lower0.7ex\hbox{$2$}}} \right)}} } \right)} \right)\& \left( {\forall ~\left( {i = 2^{r} + 1} \right)} \right)} \right.} \right\} \\ \end{aligned}$$
  When *j* = *1*:
    $$n = 1: \ll C_{1} = \left\{ {\left[ {\begin{array}{*{20}c} 1 & 0 \\ \end{array} } \right]} \right\}$$
    $$n = 2: \ll C_{2} = \left\{ {\left[ {\begin{array}{*{20}c} 3 & 2 \\ \end{array} } \right]} \right\}$$
    $$n = 3: \ll C_{3} = \left\{ {\left[ {\begin{array}{*{20}c} 5 & 4 \\ \end{array} } \right]} \right\}$$
    $$n = 4: \ll C_{4} = \left\{ {\left[ {\begin{array}{*{20}c} 7 & 6 \\ \end{array} } \right]} \right\}$$
  When concatenated $\ll C_{1} , \ll C_{2} , \ll C_{3} \& \ll C_{4}$ at the first level:
    $$\ll C_{1} \left| {\left| { \ll C_{2} } \right|} \right| \ll C_{3} || \ll C_{4} = \left\{ {\left[ {\begin{array}{*{20}c} 1 & 0 \\ \end{array} } \right]\left[ {\begin{array}{*{20}c} 3 & 2 \\ \end{array} } \right]\left[ {\begin{array}{*{20}c} 5 & 4 \\ \end{array} } \right]\left[ {\begin{array}{*{20}c} 7 & 6 \\ \end{array} } \right]} \right\}$$
  Left circular rotation of a two-row tuple on the concatenation is such that:
    $$\ll C_{3} \left| {\left| { \ll C_{4} } \right|} \right| \ll C_{1} || \ll C_{2} = \left\{ {\left[ {\begin{array}{*{20}c} 5 & 4 \\ \end{array} } \right]\left[ {\begin{array}{*{20}c} 7 & 6 \\ \end{array} } \right]\left[ {\begin{array}{*{20}c} 1 & 0 \\ \end{array} } \right]\left[ {\begin{array}{*{20}c} 3 & 2 \\ \end{array} } \right]} \right\}$$
7. The seventh row of the XOR linear space when *i* = *6* is created as follows:
  $$\begin{aligned} & R_{i} = \left\{ { \ll_{{\left[ {\left( {2^{k} } \right)C_{n} } \right]}} \left( {\sigma^{{\left( {2^{r} } \right)}} } \right){|}\forall } \right.\left( {\left( {k = \left( {{\raise0.7ex\hbox{${\left( {2^{{\left( {r - l} \right)}} } \right)}$} \!\mathord{\left/ {\vphantom {{\left( {2^{{\left( {r - l} \right)}} } \right)} 2}}\right.\kern-0pt} \!\lower0.7ex\hbox{$2$}}} \right) - 1} \right),} \right. \\ & \quad \left. {\left. {\left( {l = 1,2,3, \ldots upto\;\left( {r - l} \right) = 1} \right)\& \left( {i\;is\;even} \right)\& \left( {2^{r} < i < \left( {2^{{\left( {r + 1} \right)}} } \right)} \right)} \right)} \right\} \\ \end{aligned}$$
  First, create elements with respect to the fourth row since it is the nearest lower equivalent of 2r (i.e.,* 2*^*2*^ = *4).* Such that the row tuples created at the first level are:
    $$C_{3} ||C_{4} ||C_{2} ||C_{2} = \left\{ {\left\{ {\left[ {\begin{array}{*{20}c} 4 & 5 \\ \end{array} } \right]\left[ {\begin{array}{*{20}c} 6 & 7 \\ \end{array} } \right]\} \{ \left[ {\begin{array}{*{20}c} 0 & 1 \\ \end{array} } \right]\left[ {\begin{array}{*{20}c} 2 & 3 \\ \end{array} } \right]} \right\}} \right\}$$
  At the second/final level, the left circular rotation is with respect to the row index *i* = *2 (i.e., 2*^*(2–1)*^ = *2* = *2*^*(r−1)*^*).* Such that the elements permuted after the second/final level are:
    $$C_{4} ||C_{3} ||C_{2} ||C_{1} = \left\{ {\left\{ {\left[ {\begin{array}{*{20}c} 6 & 7 \\ \end{array} } \right]\left[ {\begin{array}{*{20}c} 4 & 5 \\ \end{array} } \right]\} \{ \left[ {\begin{array}{*{20}c} 2 & 3 \\ \end{array} } \right]\left[ {\begin{array}{*{20}c} 0 & 1 \\ \end{array} } \right]} \right\}} \right\}$$
8. The eighth row of the XOR linear space when *i* = *7* is created as follows:
  $$\begin{aligned} & R_{i} = \left\{ { \ll_{{\left[ {\left( {2^{k} } \right)C_{n} } \right]}} \left( {\sigma^{{\left( {2^{r} + 1} \right)}} } \right){|}\forall } \right.\left( {\left( {k = \left( {{\raise0.7ex\hbox{${\left( {2^{{\left( {r - l} \right)}} } \right)}$} \!\mathord{\left/ {\vphantom {{\left( {2^{{\left( {r - l} \right)}} } \right)} 2}}\right.\kern-0pt} \!\lower0.7ex\hbox{$2$}}} \right) - 1} \right),} \right. \\ & \quad \left. {\left. {\left( {l = 1,2,3, \ldots upto\left( {r - l} \right) = 1} \right)\& \left( {i\;is\;odd} \right)\& \left( {2^{r} < i < \left( {2^{{\left( {r + 1} \right)}} } \right)} \right)} \right)} \right\} \\ \end{aligned}$$
  First, create elements with respect to the fifth row since it is the nearest lower equivalent of (2r + 1) (i.e., *(2*^*2*^ + *1)* = *5)*. such that the row tuples created at the first level are:
    $$\ll C_{3} || \ll C_{4} || \ll C_{1} || \ll C_{2} = \left\{ {\left\{ {\left[ {\begin{array}{*{20}c} 5 & 4 \\ \end{array} } \right]\left[ {\begin{array}{*{20}c} 7 & 6 \\ \end{array} } \right]\} \{ \left[ {\begin{array}{*{20}c} 1 & 0 \\ \end{array} } \right]\left[ {\begin{array}{*{20}c} 3 & 2 \\ \end{array} } \right]} \right\}} \right\}$$
  At the second/final level, the left circular rotation is with respect to the row index *i* = *3 (i.e., (2*^*(2–1)*^ + *1)* = *3* = *(2*^*(r−1)*^ + *1)).* Such that the elements permuted after the second/final level are:
    $$\ll C_{4} || \ll C_{3} || \ll C_{2} || \ll C_{1} = \left\{ {\left\{ {\left[ {\begin{array}{*{20}c} 7 & 6 \\ \end{array} } \right]\left[ {\begin{array}{*{20}c} 5 & 4 \\ \end{array} } \right]\} \{ \left[ {\begin{array}{*{20}c} 3 & 2 \\ \end{array} } \right]\left[ {\begin{array}{*{20}c} 1 & 0 \\ \end{array} } \right]} \right\}} \right\}$$

The closed analysis on the XOR linear space of the order 8*8 constructed from the proposed mathematical model confirms that only the first row is computed, and the remaining rows are obtained as permutations among the values of the first row or among groups of values. Hence, the computation cost is only for the scalar additions in the first row. The proposed model is used to construct a bitwise XOR Linear space, which will be constructed well in advance of the XOR operation begins. That is, in parallel, using an idle core while reading the input to the cipher system. The computation of the proposed mathematical model, as per the time complexity based on master’s theorem, is denoted as

$$T\left( m \right) = time_{read} + time_{initialize} + time_{copy/swap}$$

That is $$T\left( m \right) = O\left( 1 \right) + O\left( C \right) + O\left( {\log_{2} i} \right)$$.

Therefore, the time complexity is $O\left( {\log_{2} i} \right)$.

The previous algorithmic approach^5^ does have the same time complexity as the proposed approach, but it is still recursive matrix generation. The second model^1^, based on Pauli’s matrix, is also a recursive matrix generation, having the time complexity $O\left( {\log_{2} \left( {\frac{n}{{2^{n} }}} \right)} \right)$.

Whereas the proposed model generates the XOR linear space row-wise using a permutation of values. That is, only the first row is initialized up to as required. The remaining are permutations of values initialized as per the mathematical Eqs. (1) to (6) as presented.

Using the proposed mathematical model, the XOR linear space is computed well in advance, as said earlier. The algorithms under study then leverage this XOR linear space to facilitate XOR-free operations. By fetching and inserting values from the XOR linear space in place of XOR operations, XOR-free operations are made easier. The operands of XOR operations are used to find the right values from the linear space. The computing cost is slightly reduced, and the algorithm runs more quickly as a result of the retrieval and placement^1^ of variables from the linear space. The proposed model is built and evaluated on the cryptographic stream cipher system SNOW 5G for the evidential demonstration of the increase in execution speed. It is necessary to investigate the implementation specifics of the planned system on SNOW 5G before addressing the speed attained by the proposed XOR-free operations. As a result, let’s begin by applying the suggested model to create the architectural designs for SNOW 5G. The proposed model is supplanted by the stream cipher technique called SNOW 5G. Actually, SNOW 5G is the advanced version of SNOW 3G. The notifiable differences between the two stream cipher techniques are given in Table 5.

**Table 5:** Key difference between SNOW 3G and SNOW 5G.

SNOW variant	Number of LFSR	Number of stages LFSR	Each stage size in bits	FSM register sizes	Output keystream
SNOW 3G	1	16	32-bit	32-bit	32-bit
SNOW 5G	2	32	16-bit	128-bit	128-bit

**Fig. 1:**
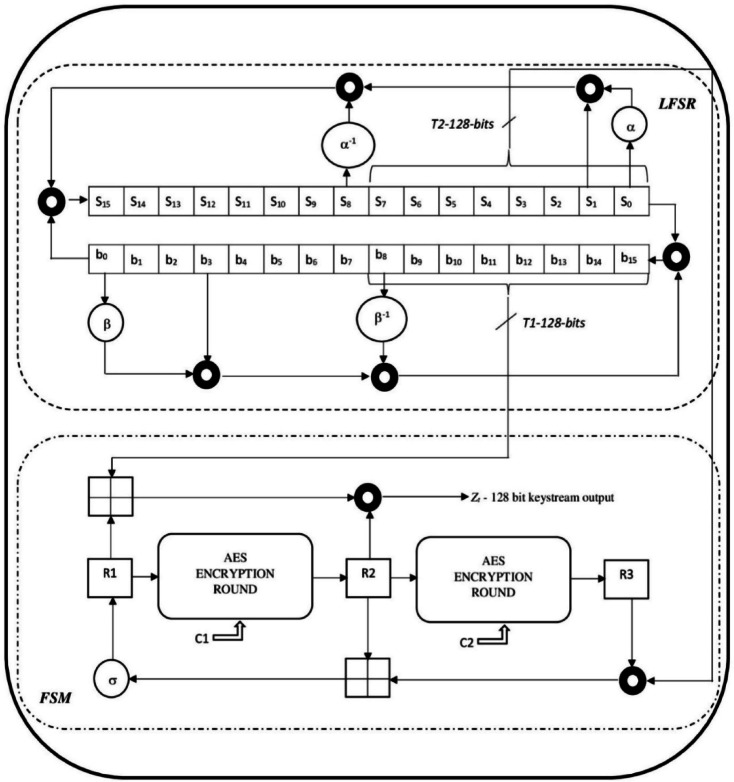
Proposed SNOW 5G architecture with XOR-free implementation.

The proposed SNOW 5G implementations using XOR-free operations are shown in the Fig. 1. The workflow of SNOW 5G is almost similar to SNOW 3G, but with a second LFSR inside the LFSR group. Each LFSR is designed using 16 registers; each is of $16$-bit in length, denoted as $\left( {S_{0} ,S_{1} ,S_{2} , \ldots ,S_{15} } \right)$ and $\left( {b_{0} ,b_{1} ,b_{2} , \ldots ,b_{15} } \right)$, so, a total of $\left( {\left( {16 - bit \times 16} \right) \times 2} \right) = 512$-bit in length. The other sub-layer, called Finite State Machine (FSM), is designed using three registers, each 128-bit in length, named as $\left( {R_{1} ,R_{2} ,R_{3} } \right)$. One more difference is the supplant of the single-round Advanced Encryption Standard (AES) in place of the substitution function of SNOW 3G. The advanced version of SNOW 5G is designed to generate a 128-bit keystream for a clock tick.

The LFSR sublayer of SNOW 5G is comprised of six XOR operations operating on 128 bits^24,25^. Similarly, six XOR operations are under the FSM sub-layer, so a total of twelve XOR operations generate every 128-bit keystream. Therefore, as the required number of keystreams increases, the required number of XOR processes/operations to be performed in SNOW 5G does increase correspondingly in the ratio 1:12. So, any modifications to enrich the speed of XOR processes/operations definitely boost overall execution of SNOW 5G. Hence, here in this work, the planned XOR-free is employed to supplant XOR by retaining its effect.

**Fig. 2:**
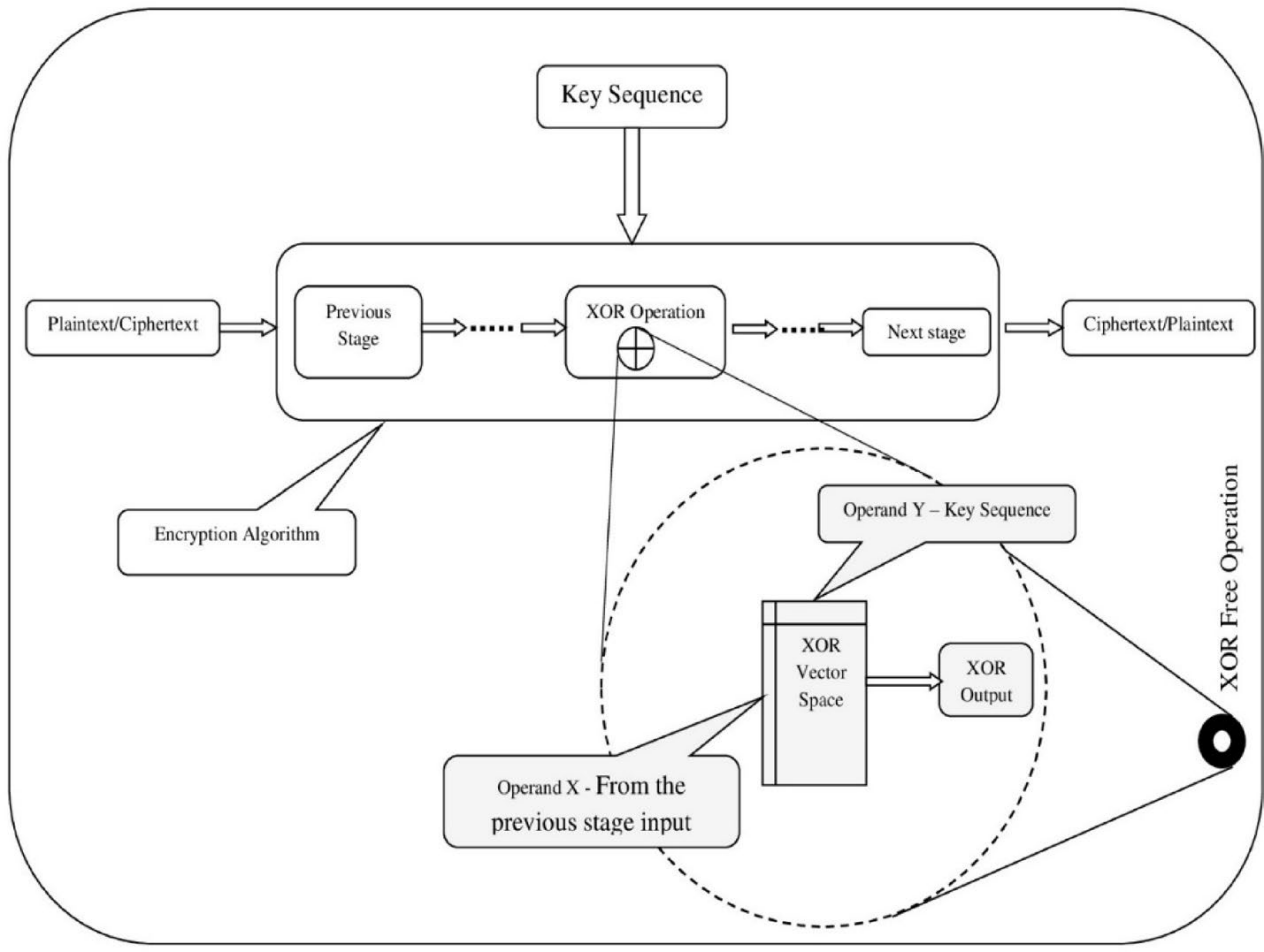
General block diagram of bitwise XOR-Free operation in a standard cipher system.

The planned XOR-free operation is represented using a symbol, “filled circular ring,” in the SNOW 5G architectural design, which is shown in Fig. 1. Whereas the computation of the filled circular ring symbol is shown in Fig. 2. The proposed designs are tested in the environment to observe the enhancement of the speed of execution, which is discussed in the next section.

The computation of the proposed bitwise XOR-Free operations in any standard cipher system is shown in Fig. 2. In this, the outcomes of every single bitwise XOR operation are replaced by XOR-free values through the bitwise XOR vector space constructed using the proposed mathematical model. The application of the same is presented in the next section.

## Application

It is very hard to come to a conclusion theoretically that the proposed method enhances the speed of execution unless analysed experimentally. Hence, an experimental setup is formulated to witness the speed enhancement of the algorithm under consideration due to the proposed model. The setup consists of Intel(R) CORE(TM) i5-7200U CPU @ 2.50 GHz 2.70 GHz, with an installed RAM of 4 GB.

Theoretically, it appears that the XOR linear space can be built in a significantly shorter amount of time. It is challenging to draw the conclusion that the speed attained by using the suggested strategy is largely devoid of experimental analysis. As a result, the implementation/application of the suggested bitwise process/operations without XOR is the only topic covered in this section. The XOR linear space is built well before its usage. That is, the linear space is ready before the XOR process begins. On multi-core systems, any idle core can be used to build the linear space while receiving information for computation. The created linear space is cached. This recorded information is positioned in the same order as corresponds to the read operands and is considered as the outcome of the XOR process/operations. This procedure is considered an XOR process/operation.

The amount of execution performance achieved as a result of the suggested XOR-free implementation is examined using the regular SNOW 5G cipher system. Hence, the linear space must be built before the SNOW 5G stream cryptography can start. An experimental setup is developed to assess the speedup achieved in the SNOW 5G stream encryption system through the suggested XOR-free employment. This configuration has 4 GB of installed RAM and an Intel(R) Core (TM) i5-7200U CPU running at 2.50 GHz and 2.70 GHz. The outcomes of the tests performed on the stream cipher system are described in relation to operational speed. The detailed descriptions of the experimental analysis conducted on the above-said environments are presented below in sequence. The speed gained through the projected model is evaluated using Eq. 7.

7 $${\text{Speed}}\;{\text{Gained}} = \left( {\frac{{{\text{T}}_{{{\text{avg}}\_{\text{standard}}}} }}{{{\text{T}}_{{{\text{avg}}\_{\text{proposed}}}} }}} \right)$$

where ${\text{T}}_{{{\text{avg}}\_{\text{standard}}}}$ denotes the average time recorded by repeatedly executing the existing/standard cipher system with XOR process/operations. ${\text{T}}_{{{\text{avg}}\_{\text{proposed}}}}$ is the average time recorded by repeatedly executing the projected cipher system through XOR-free processes/operations. Similarly, the throughput of the projected design is measured through Eq. 8.

8 $${\text{Throughput}} = \left( {\frac{{{\text{AVG}}_{{{\text{Bits}}\_{\text{processed}}}} }}{{{\text{AVG}}_{{{\text{Time}}}} }}} \right)$$

where ${\text{AVG}}_{{{\text{Bits}}\_{\text{processed}}}}$ denotes the average number of bits processed using the proposed model and ${\text{AVG}}_{{{\text{Time}}}}$ indicates the total time taken for the same bits.

The suggested technique is tested on the standard SNOW 5G cipher system, and the specifics of the experiment are presented next.

### Computation time analysis of keystream generation of SNOW 5G using planned XOR free model

SNOW 5G is an improved version of standard SNOW 3G to provide more security to the data being transmitted over the public network. A detailed description of the standard SNOW 5G is given above. Hence, in this section, it has been restricted to the experimental analysis with respect to the planned XOR-free method.

As per the study, it has been observed that the SNOW 5G has to process twelve bitwise XOR operations to generate one 128-bit keystream. So, an increase in the required count of 128-bit keystream correspondingly increases the total number of bitwise XOR operations. Hence, from the previous experimental references, it is expected that if the process is employed by means of the projected model of XOR-free in its key generation part, the execution time of SNOW 5G will be comparatively reduced.

In the experiments on SNOW 5G, initially, the standard/existing SNOW 5G is processed to generate a 128-bit keystream of a varied count. The process is repeated for each keystream count many times under consideration. The average time required for generating every keystream count of these repeated experiments is listed in the Table 6.

**Table 6:** Time difference between the proposed XOR Free SNOW 5G and the standard SNOW 5G for average Keystream Generation.

Number of keystream generated	Average key generation time for the standard SNOW 5G in seconds	Average key generation time for the proposed bitwise XOR Free SNOW 5G in seconds
655,360	221.303	217.53989
1,310,720	370.427	364.80378
1,966,080	524.667	516.71424
2,621,440	685.925	676.34073
3,276,800	855.065	842.96395
3,932,160	1036.322	1017.7726
4,587,520	1227.552	1202.4271
5,242,880	1437.003	1404.8126
5,898,240	1660.217	1618.5903
6,553,600	1895.076	1842.3569

The results show the enhancement of the speed of execution by the proposed XOR Free techniques to replace each bitwise XOR operation in the SNOW 5G’s. Upon implementing the proposed XOR-free techniques, the SNOW 5G is processed for varied keystream count in a way similar to execution carried out on standard SNOW 5G cipher systems. The average keystream generation times computed for the varied keystream count are listed in the Table 6.

**Fig. 3:**
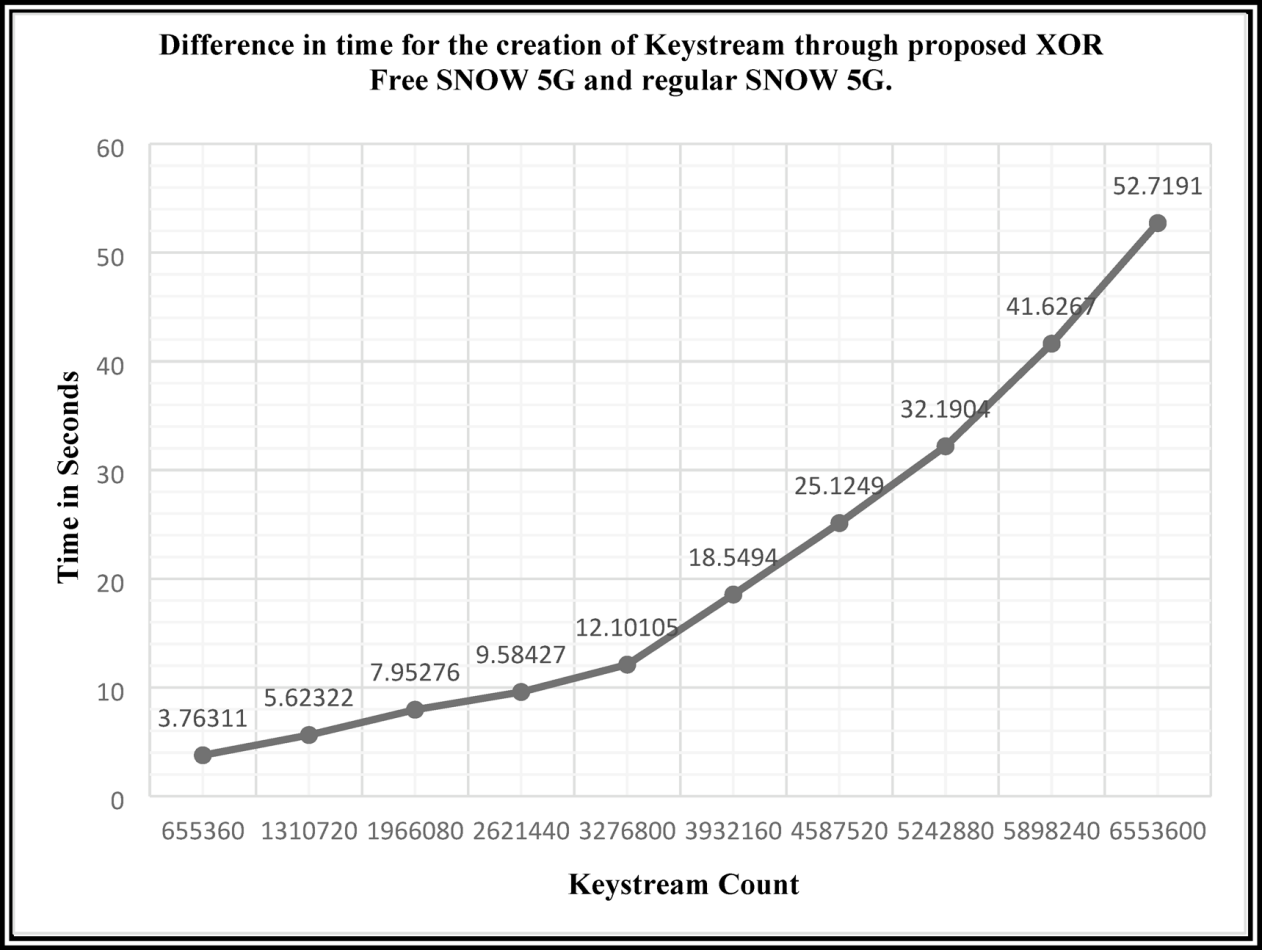
Difference in time for the creation of Keystream through proposed XOR Free SNOW 5G and regular SNOW 5G.

The proposed XOR-free employment of standard/existing SNOW 5G is seen from the trials to minimize the overall execution time. Figure 3 depicts the difference in keystream generation time between the proposed bitwise XOR Free SNOW 5G and the regular SNOW 5G. According to the experiments, the proposed SNOW 5G’s average keystream generation speedup, calculated using Eq. 7, is 1.0193**×**, while its average throughput, calculated using Eq. 8, is 459.82 Kbps.

**Fig. 4:**
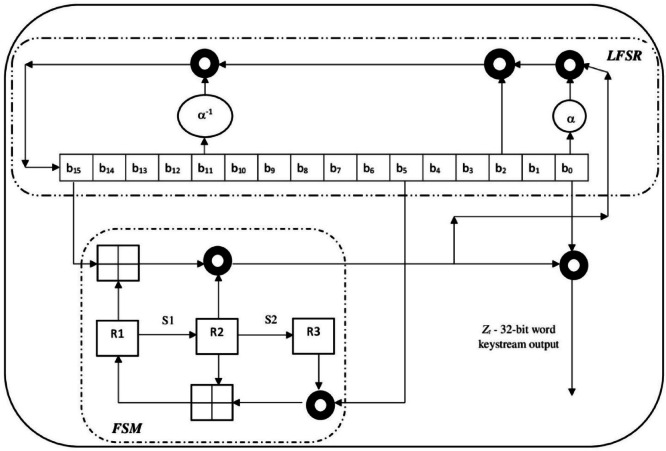
Proposed bitwise XOR-Free implementation in SNOW 3G architecture.

The SNOW 5G uses two complete Advanced Encryption Standard (AES) of 128-bit in the FSM round, and the operations are on 128-bit data. But when analysed, Dodmane et al.^1^ have worked on SNOW 3G, which operates/processes on 32-bit and generates a 32-bit keystream at a time. Both the LFSR and FSM rounds operate on 32-bit data, and the enhanced speed was 1.36×, and the through was 21.99 Mbps. Hence, to verify the reasons for the increased speed of execution in SNOW 3G compared to the SNOW 5G, the SNOW 3G is implemented using the proposed model and analysed using the same experimental setup. The implementation details of SNOW 3G are described above; hence, only a pictorial representation of the XOR-free implementation is shown in Fig. 4. The experimental details are presented below.

Figure 4 shows the replacement of bitwise XOR operations by the proposed XOR-free operations using the same filled circular ring, three each in the LFSR and FSM. The details of the keystream generation and the respective time taken using the SNOW 3G are presented in Table 7.

**Table 7:** Time difference between keystream generation and the respective time taken using the SNOW 3G.

Number of keystream generated	Average keystream generation time for the existing SNOW 3G in seconds	Average keystream generation time for the proposed bitwise XOR Free SNOW 3G in seconds
2,621,440	4.01167	2.990872
5,242,880	8.001245	6.092341
7,864,320	11.998219	9.891639
10,485,760	17.142907	14.952908
13,107,200	23.982657	19.651782
15,728,640	30.100693	24.291312
18,350,080	36.860389	27.912831
20,971,520	44.960234	31.425293
23,592,960	52.847868	35.358362
26,214,400	62.837644	39.279452

The experiments on standard SNOW 3G are carried out to analyse the speed of computation for the keystream generation using the proposed bitwise XOR-Free operations. In the experimental analysis, initially, the standard SNOW 3G is processed to determine the execution time to generate $2621440$ keystream, each of length $32$ bits. The number of keystreams $2621440$ is equivalent to $\left( {2621440 \times 32} \right)$ bits. That is $10$ MB. Similarly, the standard SNOW 3G is processed to compute the average execution times required to generate other key streams as specified in Table 7. The recorded average execution times based on the experiments carried out on the standard SNOW 3G cipher system are listed in Table 7.

Upon recording the average execution time for generating varied keystream counts, standard SNOW 3G is modified by the proposed bitwise XOR-free operations. The same experiments are carried out on the proposed bitwise XOR Free SNOW 3G.

**Fig. 5:**
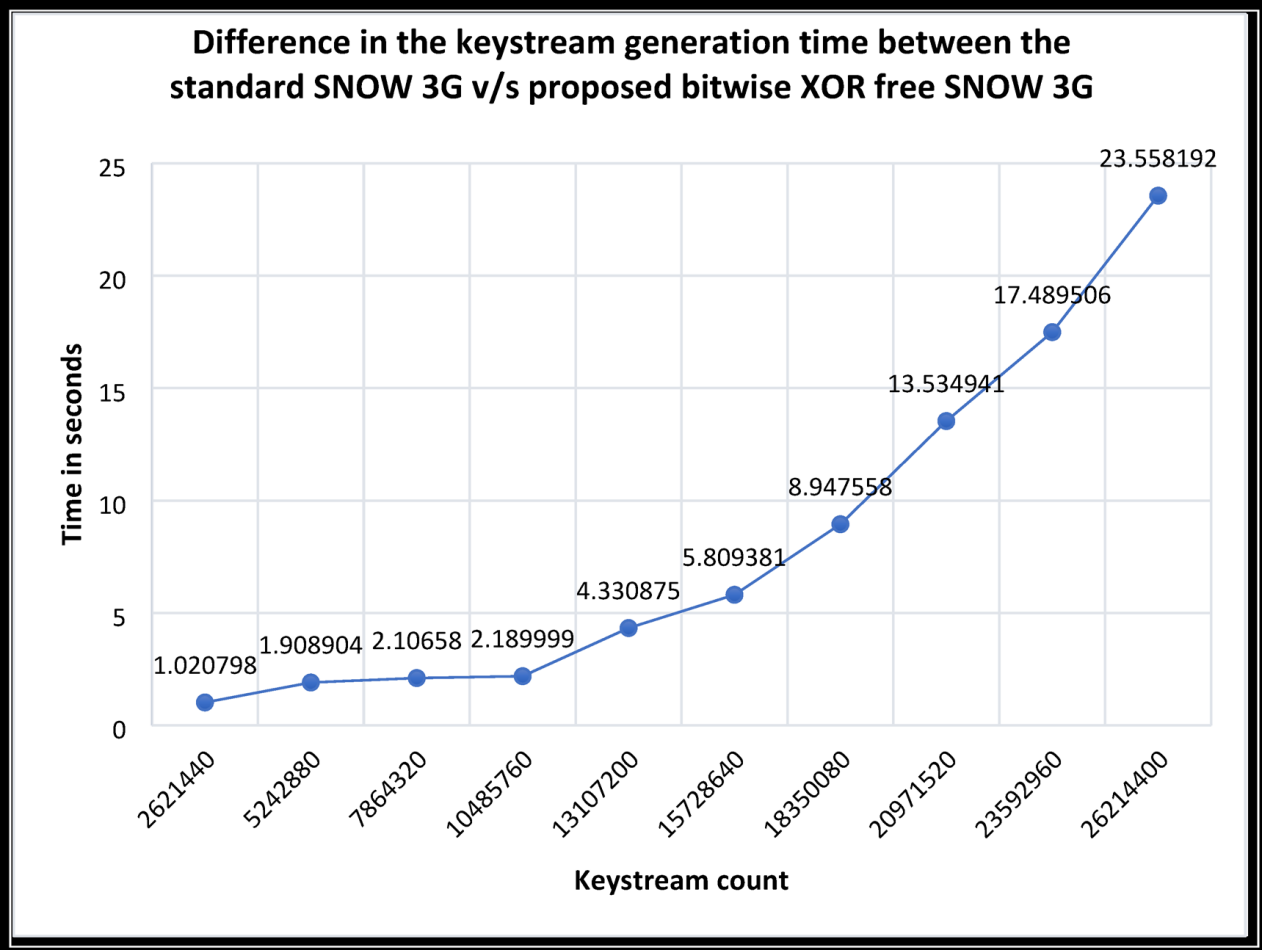
Difference in keystream generation time between the standard SNOW 3G v/s proposed bitwise XOR-Free SNOW 3G.

From the experiments, it is observed that, for the generation of the key, the proposed bitwise XOR Free SNOW 3G requires less time compared to the standard SNOW 3G. Table 7 indicates that the required number of 32-bit keystream count increases, the difference between the execution time of the proposed bitwise XOR Free SNOW 3G and the standard SNOW 3G increases correspondingly. This increased difference is shown in Fig. 5.

The graph shown in Fig. 5 indicates that as the count of the keystream generation increases, the difference in the execution time also increases exponentially. From the observation, it is obvious that the speed of computation in the proposed bitwise XOR Free SNOW 3G is improved effectively. The average keystream generation speedup of the proposed SNOW 3G, as per the Eq. (7), is 1.381864×, whereas the average throughput as per Eq. (8) is 23.659 Mbps.

From the experimental results on SNOW 3G and SNOW 5G, it is observed that the proposed XOR-free implementations has considerably increases the overall computation speed. But when computation speeds are compared between the SNOW 3G and SNOW 5G due to XOR-free operations, it is found that SNOW 3G has a greater boost. This increased computation speed in SNOW 3G is because of the size input operands. A repeated experimental analysis confirmed that if the bit size of the input operand increases, than the computation speed will slightly effected but noticeably higher than the existing methods. Hence, from the experimental analysis, it is proved that the computation speed can be enhanced using the proposed XOR-free implementations.

In the proposed method, the XOR linear space is generated by the permutation of values. Only at the beginning, it is required to initialize the values for the first row of the LUT/XOR linear space as required by the algorithm under consideration. Values for the remaining rows are generated by permuting the values of the first row depending on the row index. The XOR linear space is created in parallel using an idle core during the initial process of reading the inputs for the algorithms. The energy required for the creation of XOR linear space is negligible, as it is only copy operations. But in FGPA implementation, every XOR operation requires very high energy. This paper is restricted only with respect to the speed of execution; the experimental analysis of energy consumption is not incorporated.

To facilitate XOR-free operations, it is required to store all the results of XOR operations well in advance. The XOR linear space is a square matrix, the theoretical space complexity of creating the same is ***O(n***^***2***^***).***

From the experimental demonstrations, the results obtained by applying the proposed approach are acceptable when tested using the above-mentioned environment. Additionally, since this bitwise XOR-free version only involves retrieving and placing data from the caches^11,16,26^, it uses less power. Because the linear space is stored in the cache prior to the process/operations, resistance to side-channel assaults is increased. Considering that it prevents the cryptanalyst from accessing the linear space. Another finding is that the unit period needed for XOR-free process/operations is roughly fixed because of fetch and place from linear space. This raises the resistance against timing attacks even more^1,3,5^. Because of this, the suggested XOR-free approach offers resistance to attacks along with the speed up in executions. Therefore, it is best used in cryptography.

### Energy and memory access considerations

Although this study primarily focuses on execution speed, it is important to consider the theoretical implications of the proposed XOR-free method on energy and memory usage. Traditional XOR operations, especially when executed repeatedly in cryptographic algorithms, involve frequent memory access and CPU cycles, which can lead to increased energy consumption. In contrast, the proposed method constructs a precomputed XOR linear space that is accessed via simple fetch-and-place operations. These operations are typically less energy-intensive than arithmetic logic unit (ALU) computations, particularly in systems where memory access latency is optimized through caching.

From a memory perspective, the XOR linear space is a square matrix of space complexity O(n^2^), where *n* is the number of distinct operand values. While this introduces a higher memory footprint compared to on-the-fly XOR computation, the trade-off is justified by the reduction in dynamic power consumption and the potential for parallel access using idle cores. Moreover, since the linear space is constructed once and reused, the amortized energy cost per XOR operation is significantly reduced. This makes the approach particularly suitable for energy-constrained environments like embedded systems or mobile devices, where predictable memory access patterns can also enhance resistance to side-channel attacks.

A summary comparing the proposed XOR-free method with existing techniques, such as Free-XOR and FlexOR, is provided in Table 8, highlighting trade-offs in speed, memory usage, and security.

**Table 8:** Comparative analysis of XOR-free techniques with respect to speed, memory requirements, and security features.

Technique	Speed improvement	Memory usage	Security assumptions
Free-XOR^11^	Up to 4× in garbled circuits	Minimal (no ciphertexts for XOR gates)	Requires circular security and correlation-robust hash functions
FleXOR^12^	Up to 30% smaller circuits than Free-XOR	Slightly higher than Free-XOR (0–2 ciphertexts per XOR gate)	Requires only related-key security, weaker than Free-XOR’s assumptions
Proposed permutation-based XOR-Free	1.38× (SNOW 3G), 1.0193× (SNOW 5G)	Requires precomputed XOR linear space (O(n^2^))	No cryptographic assumptions; relies on deterministic permutations and caching

**Fig. 6:**
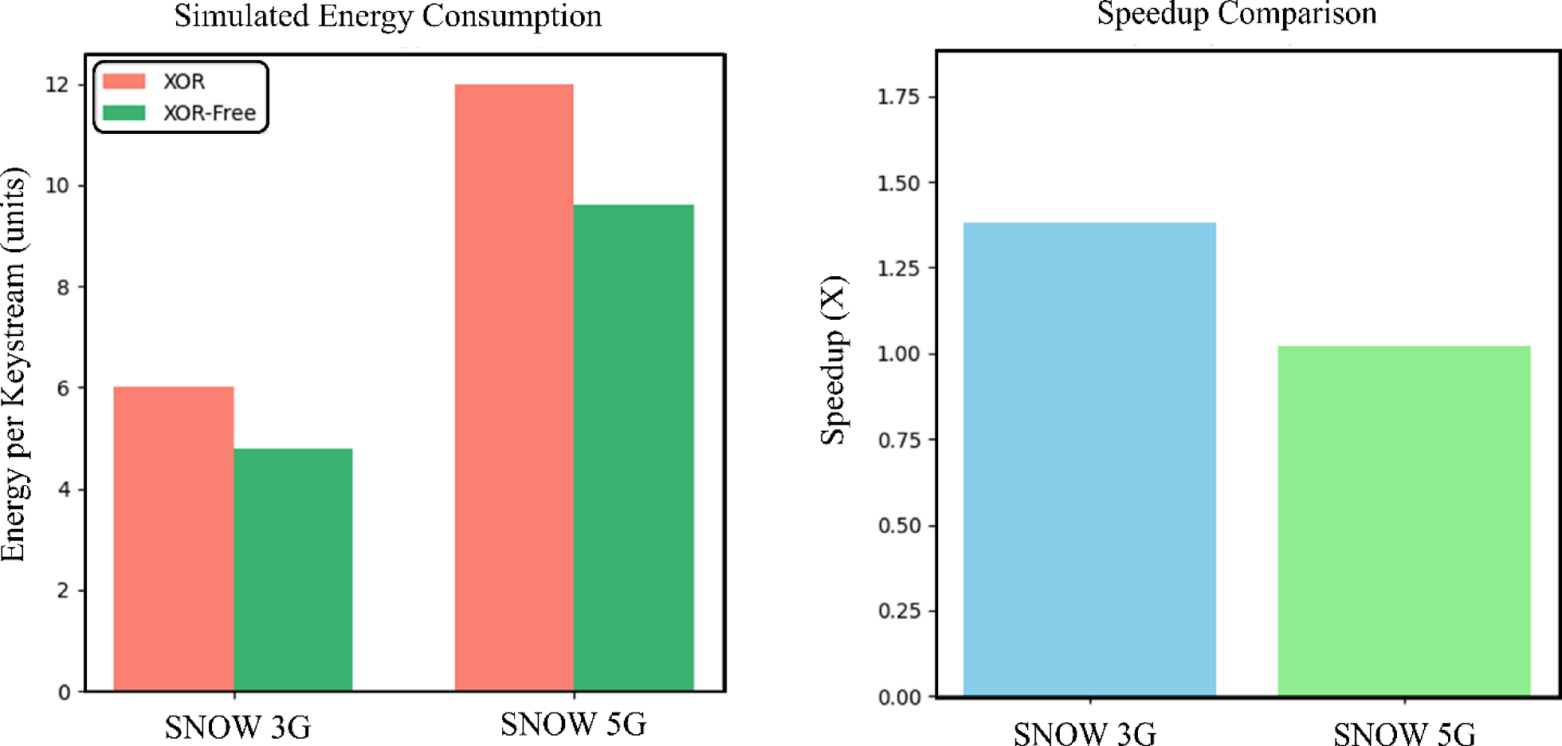
Comparison of speedup and simulated energy consumption between SNOW 3G and SNOW 5G using the proposed XOR-free method.

Figure 6 depicts the comparison of speedup and simulated energy consumption between SNOW 3G and SNOW 5G using the proposed XOR-free method. The left chart shows that SNOW 3G achieves a 1.38× speedup, while SNOW 5G achieves a 1.0193× speedup. The right chart simulates energy consumption assuming each XOR operation consumes 1 unit of energy, and XOR-free operations consume 20% less. This demonstrates the potential energy savings and performance benefits of the XOR-free approach.

## Conclusion

The linear space is a permutation of values that represents the output of the XOR process/operations accomplished among two operands. When verified on a Core i5 arrangement as described before, the linear space building time is found to be extremely short. For the SNOW 5G stream cipher system, this linear space has been used to implement bitwise XOR-free algorithms and analyse the speedup obtained. According to the suggested XOR-free implementations on SNOW 3G and SNOW 5G, a total speedup of 1.38× and 1.0193**×**, respectively. Whereas the throughput for SNOW 3G is 23.659 Mbps and for SNOW 5G is f 459.82 Kbps were realized. The increase in the computation speed in SNOW 3G is comparatively much greater to SNOW 5G. Because SNOW 5G is designed to operate and generate 128-bits data, whereas SNOW 3G is designed for 32-bit data.

Based on the findings, bitwise XOR-free strategies to reduce overall computing time are acceptable. The speedup provided by the suggested method is crucial to the cryptographic process, particularly in light of side-channel assaults. It is useful in computations as well, where a lot of XOR processes/operations are in place.

The current study focuses on the SNOW 3G and SNOW 5G stream cipher families. However, the proposed XOR-free approach is modular and can be adapted to other cryptographic systems that heavily rely on XOR operations. Many block ciphers, such as AES variants, hash functions, and authenticated encryption schemes, extensively utilize XOR in their diffusion layers and key mixing processes.

Additionally, in the context of post-quantum cryptography (PQC)—where algorithms like lattice-based and code-based cryptosystems may not rely on XOR in the same way—side-channel resistance and predictable execution timing are still critical. Since the XOR-free method uses fixed-time memory fetch and place operations, it may help harden implementations against timing attacks and power analysis, which are often more viable on PQC due to their computational complexity. Therefore, with appropriate adaptation, the permutation-based XOR-free framework proposed here has promising potential for application in broader cryptographic domains, including emerging PQC standards.

## Data Availability

All the data used in the article have been made available in the present article. The data that support the findings of this study are available from the corresponding author, Nagaraja upon reasonable request.
